# Weighted GBLUP in Simulated Beef Cattle Populations: Impact of Reference Population, Marker Density, and Heritability

**DOI:** 10.3390/ani15081118

**Published:** 2025-04-12

**Authors:** Le Zhou, Lin Zhu, Chencheng Chang, Fengying Ma, Zaixia Liu, Mingjuan Gu, Risu Na, Wenguang Zhang

**Affiliations:** 1 College of Animal Science and Technology, Inner Mongolia Agricultural University, Hohhot 010018, China; zxcvbnm8880314@163.com (L.Z.);; 2 Key Laboratory of Animal Genetics, Breeding and Reproduction of the Inner Mongolia Autonomous Region, College of Animal Science and Technology, Inner Mongolia Agricultural University, Hohhot 010018, China

**Keywords:** genomic selection, wGBLUP, selection method, estimate genomic breeding values

## Abstract

Genomic selection (GS) is a technique that integrates genomic data, pedigree information, and individual phenotypes to enhance genetic improvements of economically important traits in livestock. While it has shown significant effects in dairy cattle, its efficacy in beef cattle is lower due to breed diversity and differences in reproductive structures. Therefore, this study evaluated the impact of heritability levels, marker densities, and assessment methods (such as pedigree-based BLUP, genomic BLUP, and weighted genomic BLUP) on genomic prediction accuracy across multiple beef cattle breeds through simulations. Three beef cattle populations were simulated with heritability levels set at 0.3, 0.5, and 0.7 and marker densities set at 50 k and 770 k. The results showed that the predictive accuracy of PBLUP and GBLUP increased with higher heritability and larger reference populations. Increasing the marker density also improved the accuracy of genomic predictions; even a low marker density (50 k SNP) can significantly enhance the accuracy of genetic evaluation, although the size of the reference population needs to be optimized according to population structure, heritability, and the genetic architecture of the trait. Overall, integrating pedigree, genomic, and weighted SNP information can significantly improve the precision of GEBV prediction and reduce bias. In particular, the wGBLUP method demonstrated an improvement in the prediction accuracy of low-heritability traits in small but high-density marker populations.

## Simple Summary

Genomic selection (GS) enhances breeding efficiency by integrating genomic data with pedigree information and phenotypes. Its effectiveness varies among livestock, with beef cattle facing challenges due to breed diversity. Therefore, this study aims to evaluate the impact of different levels of heritability, marker densities, and selection designs on the accuracy of genomic prediction in multiple beef cattle breeds through simulation studies, comparing the predictive accuracy of different methods such as PBLUP, GBLUP, and wGBLUP in simulated populations, with the goal of improving the accuracy of GP in beef cattle across different genetic backgrounds. Ultimately, we found that the use of the wGBLUP method can significantly enhance the accuracy of GP. These findings are crucial for optimizing GS in beef cattle breeding.

## 1. Introduction

In the field of animal breeding, the estimation of breeding values (EBVs) typically relies on phenotypic data obtained from half-sib, full-sib, and progeny testing. However, inaccuracies in pedigree information, such as errors or omissions in individual identification, can lead to a loss of accuracy in EBV predictions [1]. Despite this, genetic improvements in livestock have progressed to the point where all available phenotypic and pedigree information can be used for evaluations, with the best linear unbiased prediction (BLUP) method calculating EBVs by correcting for environmental effects on both pedigree and phenotype [2]. This has significantly propelled genetic improvements. However, pedigree-based genetic evaluations often overlook Mendelian sampling effects, which can lead to the over- or underestimation of EBVs within families [3,4]. Sullivan emphasized that EBV assessments that do not account for Mendelian sampling can result in biased outcomes, which, in the long term, can undermine the effectiveness of genetic improvements and potentially increase the risk of unidentified deleterious genetic variations [5]. Fortunately, with the advancement of genomic technologies, particularly the development of genomic selection (GS), we are able to estimate genomic breeding values (GEBVs) more accurately by analyzing phenotypes and genotypes of single-nucleotide polymorphisms (SNP) spread across the genome [6,7]. The GS method can more truthfully estimate the kinship between individuals, and with the reduction in genotyping costs, GS is being increasingly applied in the livestock industry, significantly enhancing the accuracy of animal breeding [8]. Studies have shown that even in the presence of pedigree errors, using genomic information can improve the accuracy of GEBVs, as observed in research on Hanwoo cattle [9]. By applying GS to livestock populations, we can achieve more precise breeding value estimations for each individual, enabling more refined individual selection.

Genomic selection (GS) has become a powerful tool for improving the genetic gains of economically important traits in livestock breeding, thanks to the development of high-throughput and cost-effective genotyping technologies. By combining genomic data, pedigree information, and individual phenotypic performance, GS can accurately estimate individual genomic estimated breeding values (GEBVs), thereby enhancing the genetic gains of economic traits in livestock breeding. The predictive accuracy of GEBVs is crucial for livestock genetic evaluations, but its precision is influenced by various factors, including prediction methods [10,11,12], training population size [3,13], heritability [14,15], and marker density [16]. Although GS has achieved significant success in dairy cattle breeding, its implementation still faces challenges in other livestock such as beef cattle, due to differences in breed diversity, reproductive structure, and economic characteristics. Therefore, innovative methods are essential for the evolution of animal breeding. In livestock genetic evaluation studies, research has been conducted on three models—GBLUP, wGBLUP, and BayesR—to enhance the genomic prediction accuracy of Hanwoo carcass traits using gene expression and whole-genome sequence information. wGBLUP optimizes predictions by assigning weights to each SNP in the GRM based on its effect estimate, but studies have found its role in enhancing predictive accuracy to be limited. This may be due to the insufficient association between the preselected SNP and the causal variants that actually control the traits [17]. Zhao et al. [18] constructed a weighted GRM considering the heterogeneity of the minor allele frequency (MAF) of SNPs across different populations in large white pigs, and the results showed that wGBLUP could enhance the accuracy of joint genomic predictions for small populations with different genetic backgrounds, but its advantage diminished in large populations due to increased genetic diversity. Romé et al. [19] compared the accuracy of four BLUP models in predicting the breeding values for body weight in commercial broilers and found that the wGBLUP model, which uses GWAS information to weight SNPs, could improve accuracy by 2% to 7% over the GBLUP model in some cases. However, due to the complexity of genetic structure and the accuracy of SNP effect estimation, this advantage of the wGBLUP model is not always significant. Furthermore, in genetic evaluation studies of aquaculture species, Song et al. found that wGBLUP improved the predictive accuracy by an average of 1.5% compared to traditional GBLUP methods across four aquaculture species, demonstrating its potential to enhance genomic prediction accuracy. From these studies [20], it is shown that the advantage of wGBLUP mainly lies in assigning weights to each SNP in the genomic relationship matrix to optimize predictions, potentially increasing predictive accuracy. Although the wGBLUP method has significant theoretical advantages, its performance still varies across different cattle populations or the improvement is not significant, especially in populations with complex genetic backgrounds and environmental factors, possibly due to the following: (1) the scale and structure of the reference population, which may limit the model’s accuracy; and (2) the estimation of SNP effects, which may be influenced by the population structure and genetic background, potentially limiting the advantage of the weighted approach. Although wGBLUP provides a way to improve the predictive accuracy, its effectiveness is influenced by various factors, and further research is needed to optimize its application. Therefore, by comparing the differences in prediction accuracy across different reference population sizes, heritability levels, and chip densities in various beef cattle populations, we further conducted a comparative analysis of the differences in prediction accuracy among different beef cattle populations under various scenarios.

Genomic selection plays a central role in genetic improvement, and its accuracy is influenced by various factors such as genetic architecture, marker density, and kinship. Through simulation studies, we can rapidly assess the impact of these factors on the accuracy of genomic selection under low-cost and repeatable conditions, especially for the assessment of long-term selection effects, which is difficult to achieve with real data. Simulation studies have revealed the key role of core animals in genomic selection and how it can improve the accuracy of GS when data are limited. For instance, genomic predictions in multi-breed and purebred cattle [21], the accuracy of genomic selection in populations [22], and comparative studies of different genomic selection methods provide a scientific basis for improving the accuracy of breeding objectives and economic benefits [23]. Therefore, the main objectives of this study are as follows: (1) to investigate the effects of different levels of heritability, marker densities, and selection designs on the accuracy of genomic predictions in different beef cattle breeds, (2) to evaluate the predictive accuracy of various methods (including PBLUP, GBLUP, and wGBLUP) in simulated populations, and (3) to determine the optimal reference population size for genomic selection in different beef cattle breeds. We aim to reveal the performance differences of the wGBLUP method across different cattle populations and identify the key factors for optimizing genomic predictions by comparing different reference population sizes, heritability levels, and marker densities.

## 2. Materials and Methods

### 2.1. Data Simulation

Three different beef cattle breeds were simulated using QMSim [24]. In most reported simulation studies, just five repetitions of the simulation were conducted due to computational time and storage requirements. The initial historical populations consisted of 10,000, 5000, and 1000 individuals, respectively, and evolved over 1000 generations. At the 500th generation, the population sizes were reduced from 10,000 to 1000, from 5000 to 3000, and from 1000 to 4000, respectively. Ultimately, by the 1000th generation, the population sizes stabilized at 7120 breeding individuals, with equal sex ratios, no overlapping generations, random mating, no selection, and no migration, to create an initial linkage disequilibrium (LD) and establish a mutation–drift equilibrium in historical generations. Selection designs were based on the phenotypic performance and BLUP (EBV) approaches to create the breeds (i.e., recent populations) or distant lines from this population. Each dam had only one progeny. Within each breed, animals were randomly mated for 10 generations based on animals from the last historical generation without artificial selection, using different mating designs and replacing male and female ratios. Some breeds have different initial animal numbers, which results in slightly different breed sizes, creating different effective population sizes (Ne). To save data space without compromising computational speed, only the genotype and phenotype data of animals from generations 9 and 10 were simulated, with phenotypes derived from a standard normal distribution with a mean of 0 and variance of 1, having an overall mean as the only fixed effect. Simulations were conducted for three different beef cattle breeds, with heritability levels set at low, medium, and high, corresponding to 0.3, 0.5, and 0.7, respectively.

Based on the bovine genome assembly version ARS-UCD1.2 published by Ensembl (<https://jul2023.archive.ensembl.org/Bos_taurus/Info/Index> accessed on 7 November 2024), the 29 pairs of autosomes of the beef cattle genome were simulated with a total length of 2715.85 centimorgans (cM). The aim was to create a more realistic scenario when considering the true distances between marker numbers and QTL loci. SNP markers were uniformly distributed and randomly generated. Due to differences in the initial number of markers, two densities of diallelic sites were formed, namely 50 k and 770 k, with the number of SNPs on each chromosome band being proportional to its size. The effects of markers on traits were neutral. Across the whole genome, 25 quantitative trait loci (QTL) were evenly distributed on each chromosome, making up a total of 725 QTL. QTL effects were randomly sampled from a gamma distribution with a shape parameter equal to 0.4. Over 1000 generations of the historical population, the mutation rate for QTLs and SNPs was 2.5 × 10^5^, with an SNP mutation pattern of “recurrent”, meaning that mutations only switched between alleles without producing new mutation types. A summary of the parameters used throughout the simulation process is presented in Table 1.

**Figure 1:**
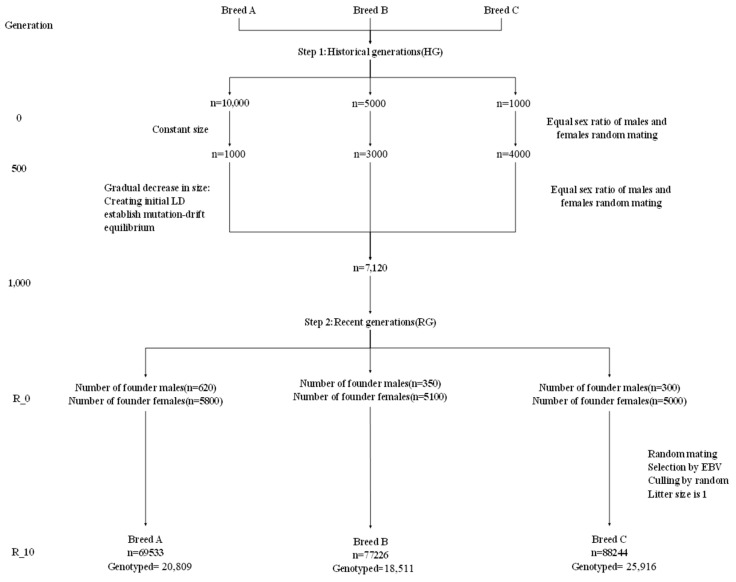
Visual presentation of the simulated data. The historical populations of 10,000, 5000, and 1000 animals were mated randomly for 1000 generations, experiencing a bottleneck and expansion phase at generation 500. Founder animals for three breeds were selected and randomly mated for 10 generations, resulting in different breed sizes and numbers of genotyped animals selected.

The genotyped animals consisted of all individuals from generations 9 and 10. The differences in the number of animals in generations 9 and 10 led to slightly different numbers of genotyped individuals per breed (Table 1). The simulation was replicated four times and the entire process is visually explained in Figure 1, including the total number of animals (genotyped or not) in generations 9 and 10 which underwent selection.

Simulate a single trait with heritabilities of 0.3, 0.5, and 0.7, each with a phenotypic variance of 1.0. The true breeding value (TBV) for each animal is calculated as the sum of the additive effects of QTLs, as follows:

(1) $${TBV}_{k}=\sum_{j=1}^{qtl}\beta_{j}\cdot Q_{kj},$$

where $\beta_{j}$ is the additive effect of ${QTL}_{j}$, and $Q_{kj}$ is the QTL genotype at locus j, coded as 0, 1, or 2, as the number of copies of a specified QTL allele is carried by an individual (k). The phenotypes (yi) were simulated by adding a residual term sampled as εi ∼ N(0,σe^2^), where σe^2^ is the residual variance.

The EBVs were estimated for all individuals in the current population from generations 9 to 10 based on phenotypic values and pedigree data. The best linear unbiased prediction (BLUP) of breeding values was obtained by Henderson’s [25] mixed linear model. The BLUP predictor has the smallest prediction error variance among all possible linear unbiased predictors. The numerator relationship matrix (A) is used in the following mixed model equations to derive BLUP of random additive effects (including polygenes and QTL):

(2) $$\left[Z^{\prime }Z+A^{-1}\frac{\sigma_{e}^{2}}{\sigma_{a}^{2}}\right]\left[\hat{a}\right]=\left[Z^{\prime }y\right],$$

where y is the vector of phenotypic records, Z is the incidence matrix relating the records to the random additive effects (a), $\sigma_{e}^{2}\;$ is the residual variance, and $\sigma_{a}^{2}\;$ is the additive genetic variance. The mixed model equations are solved by the conjugate gradient method.

Genotype data preprocessing steps are as follows. Based on PLINKv1.9 software [26], further screening of SNP was conducted with the following criteria: MAF < 0.05, genotype call rate < 0.10, individual call rate < 0.10, and Hardy–Weinberg equilibrium < 1 × 10^5^. Ultimately, after genotype quality control, a total of 58,990 and 777,962 segregating SNPs with MAF greater than 0.05 were retained for subsequent analysis. The simulation assumes that the QTL allele effects are the same across all breeds, but the frequencies of QTL vary among breeds, leading to differences in the variance for each breed. The maximum QTL variance in generations 9 and 10 did not exceed 0.02 for each breed.

### 2.2. Model and Analysis

In this study, statistical methods used to estimate breeding values include the traditional pedigree-based best linear unbiased prediction (PBLUP) method, the genomic best linear unbiased prediction (GBLUP) based on the genomic relationship matrix, and the weighted genomic best linear unbiased prediction (wGBLUP) based on genomic information.

#### 2.2.1. Pedigree-Based Best Linear Unbiased Prediction (PBLUP)

When estimating parameters using pedigree information, a mixed model is employed for PBLUP [27], which is constructed using the HIBLUP_1.4.0 software [28]. The model equation is as follows:

(3) y=Xb+Zu+e

(4) $$\left[\begin{array}{cc} X^{\prime }X & X^{\prime }Z\\ Z^{\prime }X & Z^{\prime }Z+\lambda A^{-1} \end{array}\right]\left[\begin{array}{c} \hat{b}\\ \hat{u} \end{array}\right]=\left[\begin{array}{c} X\prime y\\ Z\prime y \end{array}\right],$$

where y is a vector of the phenotype value; X contains the design matrix of the observations for fixed effects; b is the vector of the fixed effects, including sex, generation, polygene effect, and sire number; Z is the design matrix-matching phenotype value and random effect values; u is the vector of the random effects with a normal distribution; e is the vector of residual error effects with a normal distribution ~N (0, $I\sigma_{e}^{2}$); $\sigma_{e}^{2}$ is random variance; and I is the identity matrix. $\lambda =\sigma_{e}^{2}/\sigma_{a}^{2}$. A is the relationship matrix (NRM) constructed based on the pedigree information.

#### 2.2.2. Genomic-Based Best Linear Unbiased Prediction (GBLUP)

In parameter estimation using genomic information for GBLUP [29], a general linear mixed model is employed, and the model is constructed using the HIBLUP software. Although GBLUP and PBLUP use the same fixed effects, GBLUP employs a genomic relationship matrix (GRM) based on SNP markers to GEBVs. The expression for GRM is as follows [30]:

(5) $$G=\frac{MM\prime }{\sum_{i=1}^{m}2pi(1-pi)},$$

Here, M is the matrix of individual genes (where homozygotes, heterozygotes, and alternative homozygotes are converted to 0, 1, and 2, respectively), m is the total number of SNP markers, and pi is the frequency at the i-th position in the SNP.

Then, since only additive genetic effects are modeled, only these effects are considered, which are shown as follows:

(6) $$Var\left(g\right)=G\sigma_{g}^{2},$$

The general linear mixed model equation for GBLUP is as follows [31]:

(7) y=Xb+Zg+e,

(8) $$\left[\begin{array}{cc} X\prime X & X\prime Z\\ Z\prime X & Z^{\prime }Z+\lambda G^{-1} \end{array}\right]\left[\begin{array}{c} \hat{b}\\ \hat{g} \end{array}\right]=\left[\begin{array}{c} X\prime y\\ Z\prime y \end{array}\right],$$

where y is the vector of phenotypes, X is the matrix associating fixed effects with each animal individual, b is the vector of fixed effects, Z is the design matrix allocating records to genetic values, g is the vector of additive genetic effects for individuals, G is the genomic relationship matrix, e is the vector of residual error effects with a normal distribution ~N (0, $G\sigma_{e}^{2}$), and $\sigma_{e}^{2}$ is the residual variance. $\lambda =\sigma_{e}^{2}/\sigma_{g}^{2}$.

#### 2.2.3. Weighted Genomic-Based Best Linear Unbiased Prediction (wGBLUP)

In the weighted GBLUP, the model and inference are the same, except a different SNP effect vector is used when constructing the GRM. In the SLEMM-0.90.1 software [32] developed by Jiang et al., two schemes for optimizing genomic prediction by SNP weighting are provided, namely (1) based on MAF dependence of SNP effect sizes; and (2) based on the SNP effect estimates with weight W equal to the identity matrix.

SLEMM fits the following linear mixed model:

(9) $$y=X\beta +Z\alpha +e\;\alpha \sim N(0,W\sigma_{\alpha }^{2}),\;e\sim N\left(0,R\sigma_{e}^{2}\right)$$

where y is a vector of phenotypes for a quantitative trait, β is a vector of fixed effects including the mean, X is the design matrix for β, α is a vector of SNP effects with a diagonal covariance matrix $W\sigma_{\alpha }^{2}$, Z is a matrix of standardized genotypes, and e is a vector of residuals with a diagonal covariance matrix $R\sigma_{e}^{2}$. R is usually equal to an identity matrix, and the diagonal elements of W are weights, with the mean representing the relative contribution of the SNP to the genetic variance; that is, Wj represents the contribution of SNPj to the genetic variance.

Due to LD, the effect of a QTL can be captured by nearby SNP loci, and the effects of adjacent SNP tend to be similar in model fitting. Therefore, the second SNP weighting scheme is used in this study, with the formula as follows:

(10) $$W_{jj}=C\cdot \frac{1}{2S+1}\sum_{k-j-S}^{j+S}\hat{\alpha_{k}^{2}}$$

where C is a scaling constant to control the mean weight to be 1, S is the number of SNPs on each side of SNP j, and $\hat{\;\alpha_{k}}$ is the estimate of the effect of the kth SNP in an existing BLUP with W equal to the identity matrix. This specification of the jth SNP’s weight borrows information from a window of 2S+1 SNP. SLEMM first fits the model (9) with training data, where W is equal to the identity matrix, and then fits it with W computed by Equation (10).

### 2.3. Accuracy of Genomic Prediction

Because the true breeding values (TBVs) of the individuals were directly given in the simulation process, the accuracy of TBV and EBV can be directly calculated using the following formula:

Accuracy = Corr(TBV,EBV), (11)

Here, the correlation (Corr) ranges from 0 to 1, indicating the strength of the linear relationship between TBV and EBV.

## 3. Results

### 3.1. Genomic Prediction Accuracy Across All Scenarios Based on Pedigree Information

**Figure 2:**
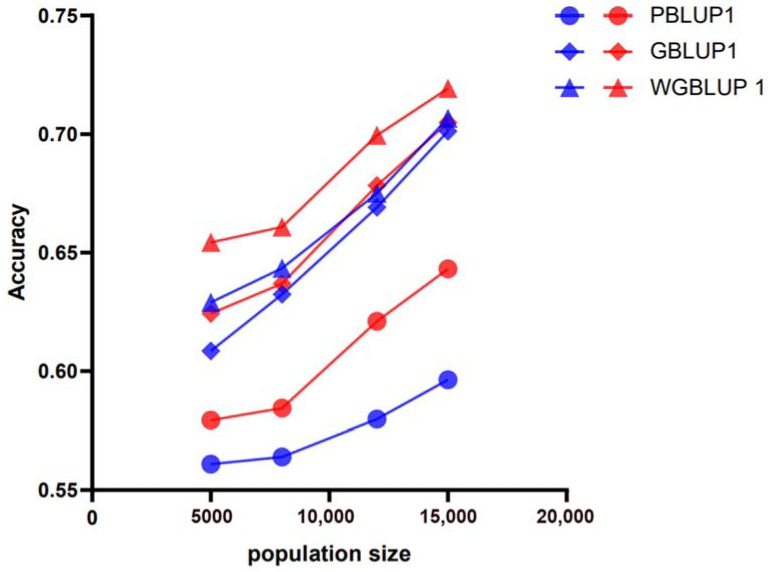
Prediction accuracy of genomic estimated breeding values (GEBVs) for simulated traits with a heritability of 0.3 in breed A using different evaluation methods: PBLUP, GBLUP, or wGBLUP. The X-axis represents the number of animals in the reference population, while the Y-axis indicates the predicted accuracy of GEBVs for the simulated traits. The blue and red lines correspond to marker densities of 50 k and 770 k, respectively.

**Figure 3:**
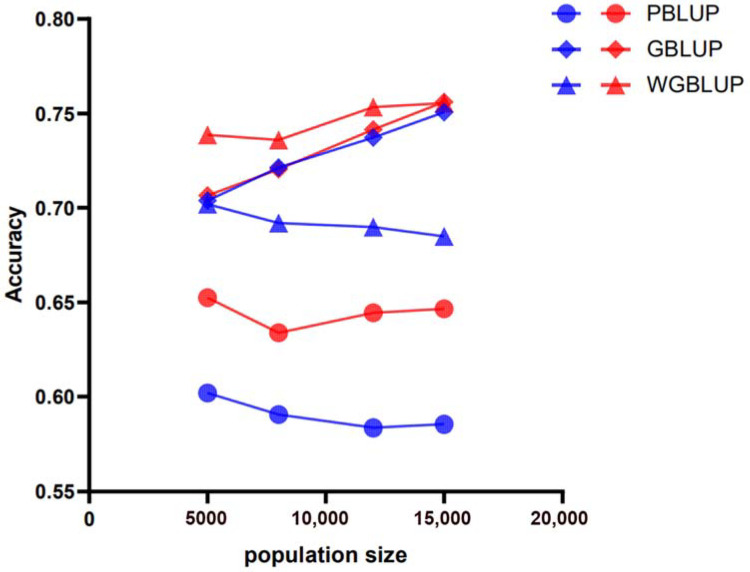
Prediction accuracy of genomic estimated breeding values (GEBVs) for simulated traits with a heritability of 0.5 in breed A using different evaluation methods: PBLUP, GBLUP, or wGBLUP. The *X*-axis represents the number of animals in the reference population, while the *Y*-axis indicates the predicted accuracy of GEBVs for the simulated traits. The blue and red lines correspond to marker densities of 50 k and 770 k, respectively.

**Figure 4:**
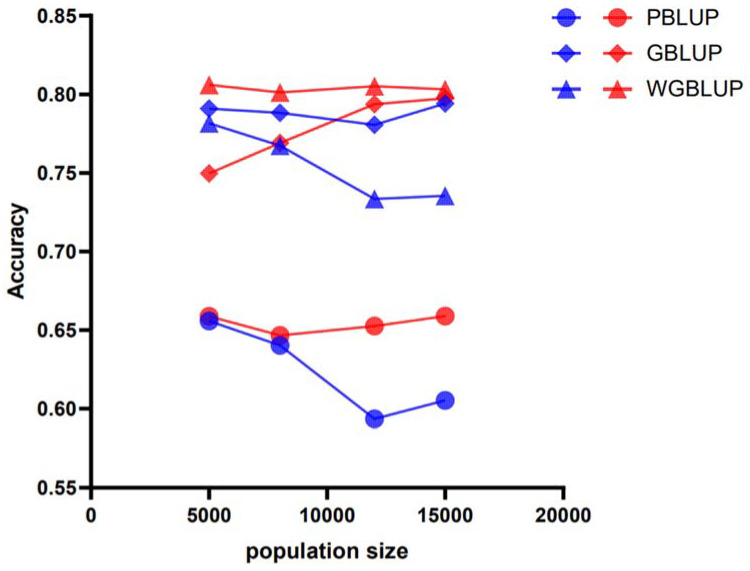
Prediction accuracy of genomic estimated breeding values (GEBVs) for simulated traits with a heritability of 0.7 in breed A using different evaluation methods: PBLUP, GBLUP, or wGBLUP. The *X*-axis represents the number of animals in the reference population, while the *Y*-axis indicates the predicted accuracy of GEBVs for the simulated traits. The blue and red lines correspond to marker densities of 50 k and 770 k, respectively.

The accuracy of genomic prediction using the PBLUP method, which is based on pedigree information, was evaluated across different scenarios (e.g., marker densities of 50 k and 770 k; heritabilities (h^2^) of 0.3, 0.5, and 0.7; and varying reference population sizes RP) for its three breeds. The PBLUP-predicted accuracies for breeds A, B, and C at a heritability of 0.3 were between 0.56 and 0.64 (Figure 2), 0.54 and 0.71, and 0.59 and 0.66, respectively. At a heritability of 0.5, they were between 0.58 and 0.65 (Figure 3), 0.67 and 0.75, and 0.61 and 0.70, respectively. At a heritability of 0.7, they were between 0.59 and 0.67 (Figure 4), 0.69 and 0.77, and 0.62 and 0.68, respectively. Compared across the three levels of heritability, a higher heritability increases the accuracy of PBLUP predictions (Supplementary Figures S1 and S2).

The prediction accuracy of PBLUP significantly increases with the enlargement of the training population (RP) size under both low and medium heritability levels, but it does not increase and even decreases under high heritability (Table 2). Under low heritability, the lowest accuracy is calculated when the training population size is 5000, while the highest accuracy is calculated when the size is 15,000 individuals. Under high heritability, the highest accuracy is calculated when the training population size is 5000, but the lowest accuracy is calculated when the size is 15,000 individuals. Secondly, the genomic prediction accuracy using pedigree information increases with the increase in marker density. For example, for breed A at a population size of 8000 and a medium level of heritability (h^2^ = 0.5), the accuracies with 50 k and 770 k marker densities are 0.5909 and 0.6338, respectively, which is an increase of 4.29%; for breed B at a population size of 12,000 and a high level of heritability (h^2^ = 0.7), the accuracies with 50 k and 770 k marker densities are 0.6956 and 0.7781, respectively, which is an increase of 8.25%; and for breed C at a population size of 15,000 and a low level of heritability (h^2^ = 0.3), the accuracies with 50 k and 770 k marker densities are 0.6162 and 0.6587, respectively, which is an increase of 4.25%. Therefore, under the PBLUP method, the prediction accuracy with a 770 k marker density is higher than that with 50 k.

### 3.2. Genomic Prediction Accuracy Across All Scenarios Based on Genomic Information

Assessments of the genomic prediction accuracy for the three breeds (A, B, and C) using the GBLUP method based on genomic information are shown at different marker densities of 50 k and 770 k, heritability (h^2^) levels of 0.3, 0.5, and 0.7, and training population (RP) sizes ranging from 5000 to 15,000 (Figure 2, Figure 3 and Figure 4). The GBLUP prediction accuracy using genomic information is higher than the accuracy of the PBLUP method using pedigree information. The prediction accuracies for breeds A, B, and C using GBLUP are from 0.61 to 0.70, 0.65 to 0.73, and 0.63 to 0.66 at a heritability of 0.3, from 0.70 to 0.76, 0.74 to 0.79, and 0.69 to 0.71 at a heritability of 0.5, and from 0.74 to 0.79, 0.78 to 0.82, and 0.70 to 0.74 at a heritability of 0.7, respectively. These results further indicate that, compared to PBLUP, the prediction accuracy of GBLUP increases by 4.77%, 6.85%, 8.95%, and 10.19% for breed A as the training population size increases from 5000 to 15,000 at a heritability of 0.3 and a marker density of 50 k (the prediction accuracies for breeds B and C also improve, but the details are not listed here).

The prediction accuracy of GBLUP also significantly increases with the enlargement of the training population (RP) size. The lowest accuracy is calculated when the training population is 5000, and the highest accuracy is calculated when the size is 15,000 individuals. In contrast, the genomic prediction accuracy under the genomic information does not increase with the increase in marker density, and even if there is an increase, it is not significant. For example, for breed A at a population size of 8000 and medium level of heritability (h^2^ = 0.5), the accuracies with 50 k and 770 k marker densities are 0.7214 and 0.7204, respectively, with a difference of 0.001; for breed B at a population size of 12,000 and a high level of heritability (h^2^ = 0.7), the accuracies with 50 k and 770 k marker densities are 0.8008 and 0.8106, respectively, with a difference of 0.0098; for breed C at a population size of 15,000 and a low level of heritability (h^2^ = 0.3), the accuracies with 50 k and 770 k marker densities are 0.6648 and 0.6643, respectively, with a difference of 0.0005. When the marker density is 50 k, the heritability is 0.7, and the training population size is 15,000, the highest genomic prediction accuracy is observed. Therefore, in GBLUP, a marker density of 50 k can demonstrate good prediction accuracy (Supplementary Table S1).

### 3.3. Genomic Prediction Accuracy Across All Scenarios Based on SNP Weighting

Assessments of genomic prediction accuracy for three breeds (A, B, and C) using the SNP-weighted wGBLUP method are shown across different marker densities of 50 k and 770 k, heritability (h^2^) levels of 0.3, 0.5, and 0.7, and training population (RP) sizes ranging from 5000 to 15,000 (Figure 2, Figure 3 and Figure 4). The prediction accuracy of wGBLUP using SNP weighting was higher than that of PBLUP, which uses pedigree information, and GBLUP, which uses genomic information. For breeds A, B, and C, the prediction accuracies using wGBLUP were from 0.63 to 0.72, 0.61 to 0.76, and 0.70 to 0.72 at a heritability of 0.3, from 0.68 to 0.76, 0.76 to 0.86, and 0.74 to 0.85 at a heritability of 0.5, and from 0.73 to 0.81, 0.79 to 0.88, and 0.77 to 0.90 at a heritability of 0.7, respectively. Under a heritability of 0.3 and a marker density of 50 k, as the training population size increased from 5000 to 15,000, the prediction accuracy of wGBLUP compared to PBLUP for breed C increased by 10.22%, 10.2%, 10.45%, and 9.64%, respectively. Compared to GBLUP, the prediction accuracy of wGBLUP under the same conditions for breed C increased by 8.92%, 7.95%, 6.51%, and 4.78%, respectively. These results further indicate that the wGBLUP method has a higher prediction accuracy than both the PBLUP and GBLUP methods (Supplementary Figures S3 and S4).

The prediction accuracy of wGBLUP does not increase significantly with the enlargement of the training population (RP) size under all scenarios, unlike PBLUP and GBLUP. When the heritability is 0.3, the prediction accuracy of wGBLUP increases significantly with the increase in RP size. However, when the heritability is 0.5 or 0.7, the prediction accuracy of wGBLUP does not show an increasing trend or it even decreases with the enlargement of RP size. For instance, with a heritability of 0.3 and a marker density of 50 k, the prediction accuracy of breed B is the lowest (0.61) when the population size is 5000, but the highest (0.73) when the population size is 15,000. With a heritability of 0.7 and a marker density of 50 k, the prediction accuracy of breed B is the highest (0.86) when the population size is 5000, but the lowest (0.79) when the population size is 15,000 (Supplementary Figures S5 and S6).

Additionally, the genomic prediction accuracy with SNP weighting increases as the marker density increases. For instance, with breed A at a population size of 8000 and medium heritability (h^2^ = 0.5), the accuracies for 50 k and 770 k marker densities are 0.6919 and 0.7360, respectively, representing a 4.41% improvement; for breed B at a population size of 5000 and a low level of heritability (h^2^ = 0.3), the accuracies for 50 k and 770 k marker densities are 0.6053 and 0.7441, respectively, indicating a 13.88% increase; and for breed C at a population size of 12,000 and a high level of heritability (h^2^ = 0.7), the accuracies for 50 k and 770 k marker densities are 0.7750 and 0.8453, respectively, which is a 7.03% enhancement. Thus, in the wGBLUP method, the prediction accuracy with 770 k markers is superior to that with 50 k markers (Supplementary Table S2).

## 4. Discussion

Due to the varying patterns of linkage disequilibrium (LD) decay across different cattle breeds, it is essential to investigate the factors affecting the accuracy of genomic predictions within diverse populations [33]. In our study, we examined the changes in the accuracy of GEBV under various selection scenarios, evaluation methods, reference population sizes, heritability levels, and marker densities, using three simulated beef cattle populations as our subjects. Breeding values are frequently employed for selecting superior individuals within a population. Among the factors considered, the evaluation method used is a potential influence on the accuracy of GEBV predictions. Across different reference population sizes, heritability levels, and marker densities, the prediction accuracy of wGBLUP was higher than that of PBLUP and GBLUP, indicating the advantage of wGBLUP over PBLUP and GBLUP. This may be attributed to the use of an SNP effect vector in the construction of the GRM, which is REML-estimated based on the stochastic Lanczos algorithm for genomic variance component estimation, thereby enhancing the accuracy of predictions through SNP weighting [34]. This approach can also be optimized for different datasets and research objectives by adjusting the window size and number of iterations. Our findings are consistent with those of Zhang et al. [35], who reported a higher prediction accuracy with wGBLUP compared to GBLUP in simulated populations.

Numerous studies have demonstrated that the size of the reference population significantly influences the accuracy of genomic selection. Generally, larger reference populations enhance the predictive accuracy of GEBVs by providing a richer source of genomic information, which allows the GRM to more accurately reflect the genetic relationships among individuals. Conversely, smaller population sizes may prevent the GRM from adequately capturing genetic variation, thereby reducing the prediction accuracy. Therefore, to improve the accuracy of GEBVs, it is advisable to use a large reference population in genomic selection whenever possible. However, considering the economic costs of whole-genome sequencing, it is necessary to find an optimal balance between population size and cost-effectiveness—the ideal reference population size. Brito et al. [36], based on previous research conclusions, used a large reference population (~15,000 animals) based on a high-density SNP chip to predict the accuracy of GEBV for growth, carcass, and meat quality traits. The results emphasized the importance of reference population size in genomic selection and the need to consider the balance between cost and prediction accuracy in practical applications. In addition, this study showed that the accuracy of genomic predictions based on PBLUP and GBLUP evaluation methods increased with the enlargement of the reference population size, which is consistent with previous genetic evaluation studies on simulated data [37], beef cattle data [27,38], dairy cattle data [39,40], and dairy goat data [41].

However, in this study, when using the wGBLUP evaluation method for accuracy prediction, the results showed that the improvement in accuracy with the increase in the reference population size was moderate and reached a plateau, even showing a trend of decline. When the reference population included 5000 animals, the GEBV accuracy was maximized for the three breeds under medium to high heritability levels, with values of 0.70 and 0.78 (A), 0.79 and 0.86 (B), and 0.79 and 0.85 (C). This trend is consistent with the results of Takeda et al. [42] who conducted a weighted assessment based on the maximum likelihood (ML) method, but the predictive accuracy we obtained was higher. The reason for this outcome might be that the ML method yields fewer correlation weight factors and less close genetic relationships between SNPs, while wGBLUP utilizes LD relationships to obtain physically proximate SNPs and makes their effect sizes similar [43]. Therefore, in the case of high heritability and large reference populations, the prediction accuracy of wGBLUP may not significantly improve and may even show a downward trend. This is likely because, in such cases, the basic GBLUP method can already provide a relatively high prediction accuracy, and the additional improvements brought by the weighting mechanism of wGBLUP are limited. Moreover, large reference populations may encompass more genetic variation, which could dilute the effects of the weighting mechanism. Although wGBLUP performs well in most scenarios, its performance enhancement may be restricted in certain specific contexts. This indicates that, in practical applications, it is necessary to select the appropriate genomic prediction method based on specific research objectives and data characteristics. Thus, the computational complexity of wGBLUP and its reliance on the genetic diversity of the training population also need to be taken into account in practical applications. Additionally, in the study by Uemoto et al. [44], which used a reference population size increasing from 200 to 1200 animals for a genetic evaluation, the accuracy of the genomic prediction did not reach a plateau. However, this study used about 13 times more animals than previous ones, indicating that under phenotype data with medium to high levels of heritability, the accuracy of genomic prediction gradually approaches a plateau. The results of this study and the aforementioned studies demonstrate that when the reference population reaches a certain level, it can effectively ensure the accuracy of genomic selection.

Genomic selection is a key strategy for improving the efficiency of genetic improvements, especially for traits with low heritability and those that are difficult to measure directly. In this study, we simulated scenarios with low, medium, and high levels of heritability to assess the effectiveness of genomic selection, and the results showed that an increase in heritability affects the accuracy of genomic prediction. Regardless of the evaluation method used, whether PBLUP, GBLUP, or wGBLUP, the accuracy of genomic prediction increases with the size of h^2^. Under different h^2^ scenarios, the accuracy of wGBLUP is higher than that of PBLUP and GBLUP. Nwogwugwu et al. [9] indicated that a higher h^2^ leads to greater predictive accuracy, as h^2^ represents the proportion of phenotypic variation caused by genetic factors, which directly affects the accuracy of EBV prediction. High heritability implies a strong correlation between phenotypic values and breeding values, thereby improving the accuracy of EBV prediction. This means there is a relationship between h^2^ and predictive accuracy, as we have observed. Many studies have proven that accuracy increases with increasing h^2^ values, which is consistent with our research findings [45,46,47]. Furthermore, Gualdrón Duarte et al. [48] mentioned that when large-effect variations contribute to complex traits, genomic prediction methods that assign higher variance to these variations can achieve a higher predictive accuracy. This implies that if a trait has high heritability, prediction models based on these genetic variations may be more accurate. Moreover, by using a weighted GRM, the predictive accuracy in the GBLUP method can be further improved. This is also consistent with our study results, in which the accuracy of wGBLUP is higher than that of PBLUP and GBLUP.

A higher marker density provides more genetic information on chromosomes of the same length, facilitating the easier identification of markers in linkage disequilibrium with QTL. Generally, as the marker density increases, the accuracy of genomic predictions also improves. Some authors have reported that the accuracy of genetic evaluations using genotype data from high-density chips is higher than that using genotype data from low-density chips [31,49]. In the article by Zhu et al. [50], different subsets of single-nucleotide polymorphisms (SNP) were constructed to estimate GEBVs, and the impact of these different densities on predictive ability was assessed. The results showed that predictive ability significantly increased with the inclusion of more SNPs up to a certain marker density (200K SNP). Brito et al. [51]’s simulation study using 50 k and 770 k marker densities demonstrated that the predictive accuracy improved with an increase in the number of markers. This is consistent with the conclusions obtained in this study using two evaluation methods, PBLUP and wGBLUP: the predictive accuracy increases with the marker density. By using high-density marker genotypes, a realized relationship matrix between individuals can be constructed, with elements representing the proportion of identical-by-descent (IBD) genome content between individual pairs, which enhances the BLUP estimation of breeding values, especially for individuals lacking direct phenotypic data, thus increasing the predictive precision. In short, dense genetic markers contribute to more accurate predictions of individual breeding potential [3].

However, the research by Solberg et al. [16] showed that as the marker density increases, the accuracy of genomic predictions improves, but the rate of improvement slows down and may stabilize after reaching a certain density. This indicates that there is a balance point between the marker density and predictive accuracy; beyond this point, the marginal benefits of increasing the number of markers on the predictive accuracy will gradually decrease. Rabier et al. [52], in their study on perennial ryegrass, demonstrated that using 3000 to 5000 evenly distributed SNP markers can achieve a genomic prediction accuracy close to that of high-density markers. Simulation analyses further revealed that when the marker density is reduced to about 1000 SNP, the predictive accuracy reaches a plateau, and additional increases in the number of markers contribute little to enhancing the predictive accuracy. Therefore, a reasonable choice of marker density can strike a balance between costs and benefits. Moreover, studies have shown that imputing missing genotypes in the 54K dataset does not significantly enhance the accuracy of genomic predictions. Although high-density marker panels should theoretically enhance the predictive accuracy, the actual improvement is modest and significantly influenced by the choice of model and data quality [53,54,55]. When applying the GBLUP method for genomic evaluation, we found that whether using 50 k or 770 k SNP marker densities, the difference in predictive accuracy becomes insignificant after reaching a certain threshold, which is consistent with previous studies. The reasons for this phenomenon may include the following: (1) when the marker density reaches a certain level, most significant LD regions have been covered; (2) after most major-effect QTL have been tagged, additional markers may only capture additional minor-effect QTL, contributing little to the overall accuracy; and (3) as the marker density increases, the LD between markers increases, leading to redundant marker information. After reaching a certain density, many markers may provide similar information about the same genetic variation, no longer providing additional improvements in predictive accuracy [16,56,57]. The use of over 500K SNP markers only brings a limited increase in accuracy, indicating that the addition of more SNPs mainly reduces the sampling error of the genomic relationship matrix G. Therefore, the primary role of genomic information is to enhance the accuracy of genetic evaluations by more precisely estimating the genetic relationships between individuals, including Mendelian sampling [58]. Even though the accuracy of predictions via the PBLUP method increases with marker density, it does not exceed the accuracy of predictions via the GBLUP method, and among these three methods, wGBLUP is the best choice for prediction accuracy. This is because wGBLUP assigns different weights to markers by considering the actual extent of the LD between the markers and QTL. This means that these methods can more effectively utilize marker information, especially at higher marker densities, and can better distinguish the importance of markers, thereby continuously improving the predictive accuracy.

In the field of genomic selection in plants and animals, numerous genetic evaluation methods such as GBLUP, ssGBLUP, wGBLUP, and Bayesian methods have been widely implemented and have significantly impacted prediction accuracy. Research has indicated that in genomic evaluations of beef cattle [30,59,60,61], dairy cattle [10], pigs [62], and simulated American mink [63], the average accuracy of GBLUP methods is markedly higher than that of PBLUP methods based on pedigree information. Although GBLUP is widely used in beef cattle genomic evaluations due to its simplicity and low computational requirements, its reliance on the LD between the markers and QTL limits its potential to capture new information and improve the prediction accuracy. Consequently, Nwogwugwu et al. [64] introduced three different weights in ssGBLUP to address the issue of multicollinearity among variables and the low-rank problem of matrices, which may render the inversion of matrices difficult or impossible. Therefore, Haque et al. [65], in their study on the genomic prediction accuracy for reproductive and carcass traits in Hanwoo cattle, demonstrated a clear advantage of wGBLUP over traditional GBLUP methods by assigning different weights to significant SNPs, highlighting the importance of incorporating heterogeneity in SNP effect sizes in genomic evaluations to enhance prediction performance. This result aligns with the comparative results observed in this study, and other studies have also shown that the accuracy of predictions via the wGBLUP method is higher than that of predictions via the PBLUP and GBLUP methods. Lourenco et al. [12], in their analysis of genomic prediction accuracy in an Israeli Holstein cattle population, found that weighted single-step GBLUP (WssGBLUP) significantly improved prediction accuracy by assigning higher weights to SNPs that have a greater impact on the target traits, especially when evaluating percentage traits, showing its significant potential to enhance the accuracy of genomic evaluations. Compared with PBLUP and GBLUP, wGBLUP considers the similar effects of physically adjacent SNPs due to the LD as weights in the model. Although there are various methods for calculating the allocation of weights, they all further optimize the accuracy of genomic predictions and even provide the flexibility to adapt to different datasets and research objectives by adjusting the window size and iteration number. Karaman et al. [66] estimated the covariance of SNP effects using Bayesian whole-genome regression methods and applied these covariances as weights in multi-trait wGBLUP methods. The results showed that when using the SNP covariances estimated in the Bayesian method as weights, wGBLUP can achieve a prediction accuracy comparable to the Bayesian method in multi-trait genomic predictions. In particular, when considering 100 adjacent SNPs as a common weight, wGBLUP ouRPerforms the traditional GBLUP method. There are also many studies on the genomic prediction accuracy of reproductive and carcass traits in Hanwoo cattle that compare various genomic evaluation methods, including the traditional GBLUP, wGBLUP, and machine learning methods. The results show that compared with the traditional GBLUP method, wGBLUP can significantly improve the prediction accuracy by assigning higher weights to SNPs with larger effects, especially for traits influenced by major genes. Additionally, the application of SNPs pre-selected based on gene expression information and GWAS results in wGBLUP further enhances the predictive power of the model. These findings emphasize the importance of considering SNP weights in genomic predictions and demonstrate the potential of wGBLUP to improve the accuracy of predictions of complex traits, especially for traits with known genetic structures and those that are influenced by a few major genes [17,67,68]. According to the above research results, the wGBLUP method has shown superior predictive performance to PBLUP and GBLUP in all cases, thereby enhancing the genetic improvement in beef cattle breeding. Future research could build on this foundation by integrating deep learning models (such as convolutional and recurrent neural networks) with wGBLUP to capture complex interactions between SNPs, thereby improving the prediction accuracy. Additionally, combining wGBLUP with Bayesian methods, using the posterior variance of SNP effects estimated by Bayesian methods as weights, can further optimize predictions, especially for traits influenced by large-effect QTL. These advancements not only enhance the prediction accuracy but also provide more powerful tools for beef cattle breeding, driving the continuous development of breeding technologies.

## 5. Conclusions

In this study, we explored the potential application of genomic selection in beef cattle breeding using simulated datasets that encompassed diverse reference populations, heritability levels, selection strategies, and marker densities. We found that even a low marker density (50 k SNP) can significantly enhance the accuracy of genetic evaluations, although the size of the reference population needs to be optimized based on the population structure, heritability, and genetic architecture of the traits. The study demonstrated that integrating pedigree, genomic, and weighted SNP information can significantly improve the precision of GEBV predictions and reduce bias. Particularly, the wGBLUP method showed an advantage in enhancing the predictive accuracy for low heritability traits in small-scale but high-density marker populations. Our research emphasizes the importance of fully utilizing genetic and population structure information in genetic evaluations and points out that by fine-tuning selection methods, evaluation strategies, reference population sizes, heritability levels, and marker densities, one can more effectively harness the genetic diversity within populations, advancing the genetic progress in beef cattle breeding. This study not only provides key technical parameters for the formulation of genomic breeding strategies in beef cattle but also establishes an optimizable framework for genomic prediction that can be extended to other livestock species. Future research can build on this foundation to further explore the integration of multi-omics data and the application of machine learning algorithms in genomic predictions, thereby continuously driving the innovative development of livestock breeding technologies.

## Figures and Tables

**Table 1:** Parameters of the simulation process.

Population Structure	A	B	C
Step 1: Historical generations (HG)			
Number of generations phase 1 (size)	0 (10,000)	0 (5000)	0 (1000)
Number of generations phase 2 (size)	500 (1000)	500 (3000)	500 (4000)
Number of generations phase 3 (size)	1000 (7120)		
Step 2: Expanded generations (EG)			
Number of founder males from HG	620	350	300
Number of founder females from HG	5800	5100	5000
Number of generations	10		
Number of offspring per dam	1		
Selection and mating	ebv/h		
Sire replacement and growth rate	0.5065 0.072	0.1851 0.1038	0.063 0.123
Dam replacement and growth rate	0.30 0.098	0.3015 0.1629	0.105 0.355
Mating system	Random		
Culling design	Random		
Genome			
Number of chromosomes	29 (no X Chr)		
Genome length	2486cM		
Number of markers	58,990 (50 k)/777,962 (770 k)		
Marker/QTL positions	Random		
Number of marker/QTL alleles	2/2 3 4		
Marker of allele frequencies	Equal		
QTL allele effects	Equal		
Mutation rate	2.5 × 10^5^		

**Table 2:** Accuracies of genomic prediction using PBLUP, GBLUP, or wGBLUP procedures under different training populations, with varying levels of heritability and marker densities for breed A.

Population Size	h^2^	50 k			770 k		
		PBLUP	GBLUP	wGBLUP	PBLUP	GBLUP	wGBLUP
5000	0.3	0.5609333	0.6085367	0.6289896	0.5793174	0.6243117	0.6543064
	0.5	0.6018924	0.703766	0.7018991	0.6524662	0.7065225	0.7386434
	0.7	0.6658209	0.7908608	0.781666	0.6588115	0.7497605	0.8060416
8000	0.3	0.5639306	0.6323918	0.6272176	0.5843689	0.6368854	0.6608198
	0.5	0.590504	0.7213553	0.6919124	0.6338298	0.7204071	0.7359905
	0.7	0.6403381	0.7882723	0.7673341	0.646572	0.7690514	0.8011734
12,000	0.3	0.5797459	0.6691037	0.6550116	0.6209803	0.6783403	0.6995511
	0.5	0.5835927	0.7372556	0.6898773	0.6443724	0.7414165	0.7533543
	0.7	0.5935911	0.7806763	0.733462	0.652524	0.7937998	0.8052261
15,000	0.3	0.5963547	0.7011606	0.6717033	0.6431027	0.7049134	0.7192473
	0.5	0.5854196	0.750636	0.684855	0.6465078	0.7560124	0.7554457
	0.7	0.6052722	0.7941701	0.7355052	0.6588932	0.7974239	0.803195

## Data Availability

The original contributions presented in the study are included in the article; further inquiries can be directed to the corresponding author.

## Supplementary Materials

The following supporting information can be downloaded at: https://www.mdpi.com/article/10.3390/ani15081118/s1, Figure S1: Prediction accuracy of Genomic Estimated Breeding Values (GEBVs) for simulated traits with a heritability of 0.3 in breed B using different evaluation methods: PBLUP, GBLUP or wGBLUP; Figure S2: Prediction accuracy of Genomic Estimated Breeding Values (GEBVs) for simulated traits with a heritability of 0.3 in breed C using different evaluation methods: PBLUP, GBLUP or wGBLUP; Figure S3: Prediction accuracy of Genomic Estimated Breeding Values (GEBVs) for simulated traits with a heritability of 0.5 in breed B using different evaluation methods: PBLUP, GBLUP or wGBLUP; Figure S4: Prediction accuracy of Genomic Estimated Breeding Values (GEBVs) for simulated traits with a heritability of 0.5 in breed C using different evaluation methods: PBLUP, GBLUP or wGBLUP; Figure S5: Prediction accuracy of Genomic Estimated Breeding Values (GEBVs) for simulated traits with a heritability of 0.7 in breed B using different evaluation methods: PBLUP, GBLUP or wGBLUP; Figure S6: Prediction accuracy of Genomic Estimated Breeding Values (GEBVs) for simulated traits with a heritability of 0.7 in breed C using different evaluation methods: PBLUP, GBLUP or wGBLUP; Table S1: Accuracies of genomic prediction using PBLUP, GBLUP or wGBLUP procedures under different training populations, with varying levels of heritability and marker densities for breed B; Table S2: Accuracies of genomic prediction using PBLUP, GBLUP or wGBLUP procedures under different training populations, with varying levels of heritability and marker densities for breed C.

## Abbreviations

The following abbreviations are used in this manuscript:

GS	Genomic selection
TBV	True breeding values
EBV	Estimation of breeding values
BLUP	Best linear unbiased prediction
GEBV	Genomic estimated breeding value
SNP	Single-nucleotide polymorphisms
MAF	Minor allele frequency
LD	Linkage disequilibrium
QTL	Quantitative trait loci
PBLUP	Traditional pedigree-based best linear unbiased prediction
GBLUP	Genomic best linear unbiased prediction
wGBLUP	Weighted best linear unbiased prediction
GRM	Genomic relationship matrix
