# Supplementary material for: Weighted GBLUP in Simulated Beef Cattle Populations: Impact of Reference Population, Marker Density, and Heritability
Source: Animals (Basel). 2025 Apr 12;15(8):1118. doi: 10.3390/ani15081118 (PMC12024408; doi:10.3390/ani15081118)
Supplement: Supplementary file 1 [file animals-15-01118-s001.zip › animals-3555133-supplementary.pdf]

Assessment of Genomic Prediction Using Weighted GBLUP Evaluation Approaches in Simulated Populations of Beef Cattle

Le Zhou[1,2], Fengyiing Ma[1,2], Zaixia Liu[1,2], Chencheng Chang[1,2], Lin Zhu[1,2], Mingjuan Gu[1,2], Risu Na[1,2], Wenguang Zhang[1,2,*]

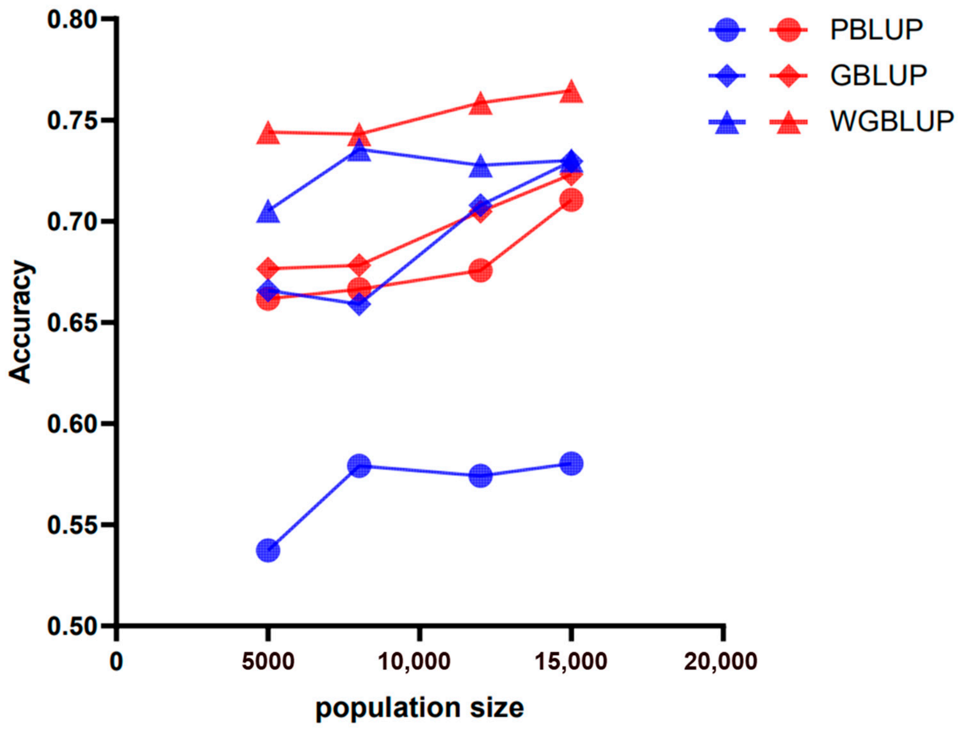

Supplementary Figure S1. Prediction accuracy of Genomic Estimated Breeding Values (GEBVs) for simulated traits with a heritability of 0.3 in breed B using different evaluation methods: PBLUP, GBLUP or WGBLUP. The X-axis represents the number of animals in the reference population, while the Y-axis indicates the predicted accuracy of GEBVs for the simulated traits. The blue and red lines correspond to marker densities of 50 k and 770 k, respectively.

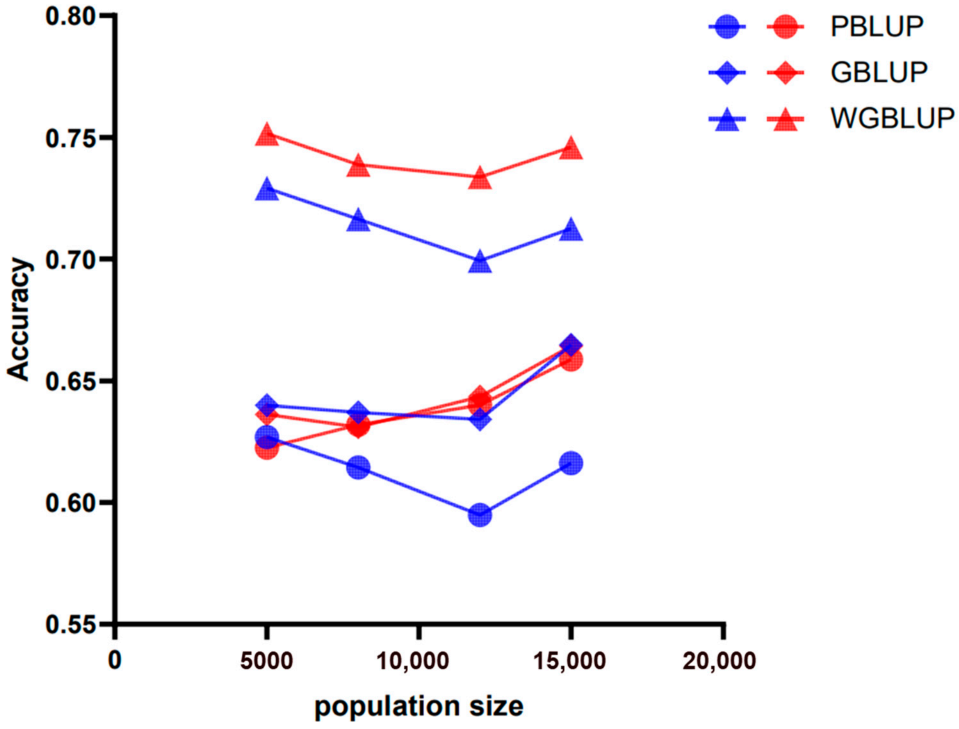

Supplementary Figure S2. Prediction accuracy of Genomic Estimated Breeding Values (GEBVs) for simulated traits with a heritability of 0.3 in breed C using different evaluation methods: PBLUP, GBLUP or WGBLUP. The X-axis represents the number of animals in the reference population, while the Y-axis indicates the predicted accuracy of GEBVs for the simulated traits. The blue and red lines correspond to marker densities of 50 k and 770 k, respectively.

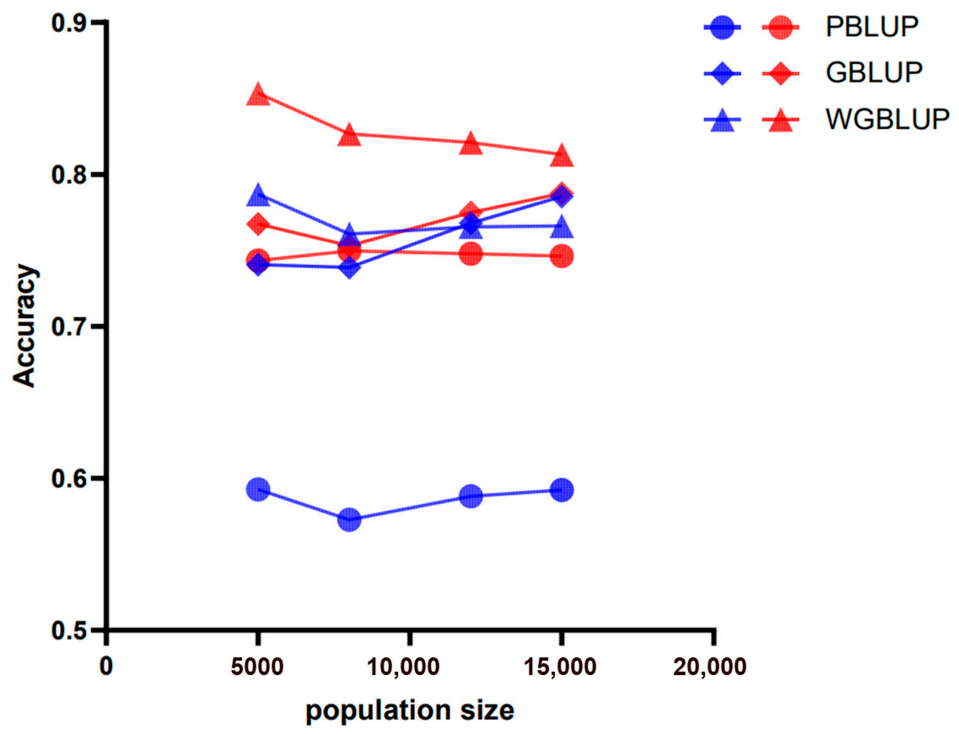

Supplementary Figure S3. Prediction accuracy of Genomic Estimated Breeding Values (GEBVs) for simulated traits with a heritability of 0.5 in breed B using different evaluation methods: PBLUP, GBLUP or WGBLUP. The X-axis represents the number of animals in the reference population, while the Y-axis indicates the predicted accuracy of GEBVs for the simulated traits. The blue and red lines correspond to marker densities of 50 k and 770 k, respectively.

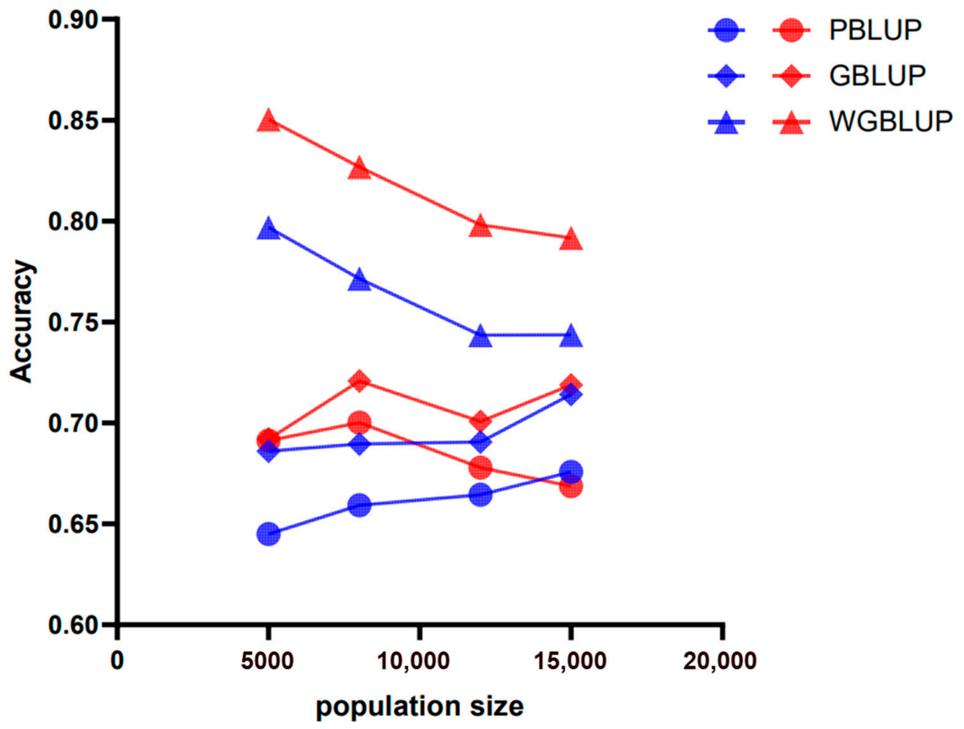

Supplementary Figure S4. Prediction accuracy of Genomic Estimated Breeding Values (GEBVs) for simulated traits with a heritability of 0.5 in breed C using different evaluation methods: PBLUP, GBLUP or WGBLUP. The X-axis represents the number of animals in the reference population, while the Y-axis indicates the predicted accuracy of GEBVs for the simulated traits. The blue and red lines correspond to marker densities of 50 k and 770 k, respectively.

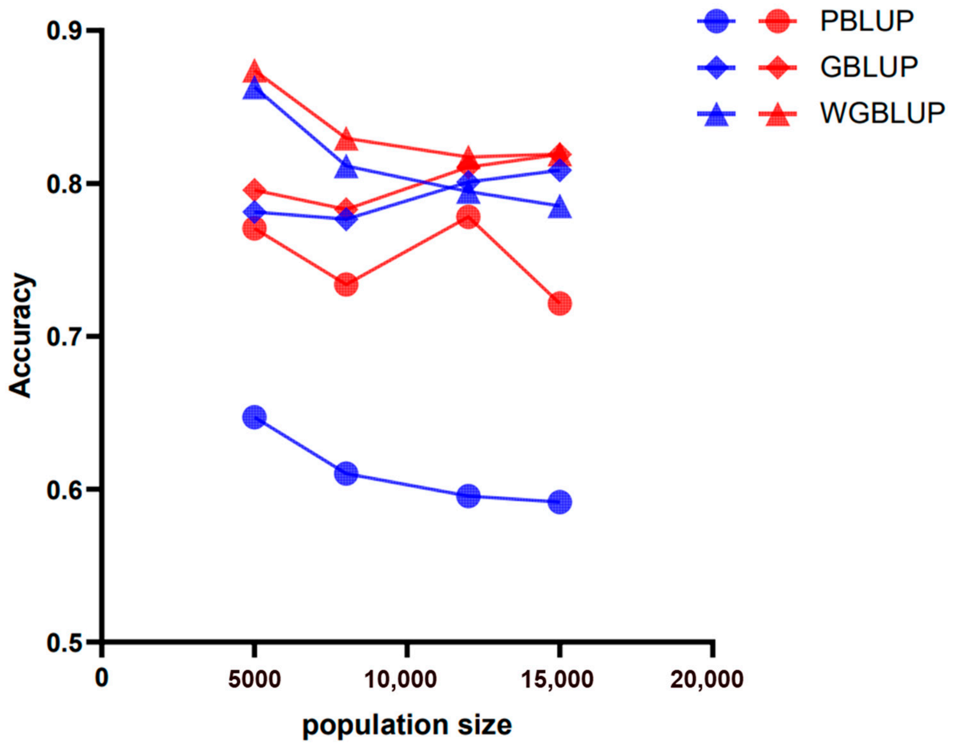

Supplementary Figure S5. Prediction accuracy of Genomic Estimated Breeding Values (GEBVs) for simulated traits with a heritability of 0.7 in breed B using different evaluation methods: PBLUP, GBLUP or WGBLUP. The X-axis represents the number of animals in the reference population, while the Y-axis indicates the predicted accuracy of GEBVs for the simulated traits. The blue and red lines correspond to marker densities of 50 k and 770 k, respectively.

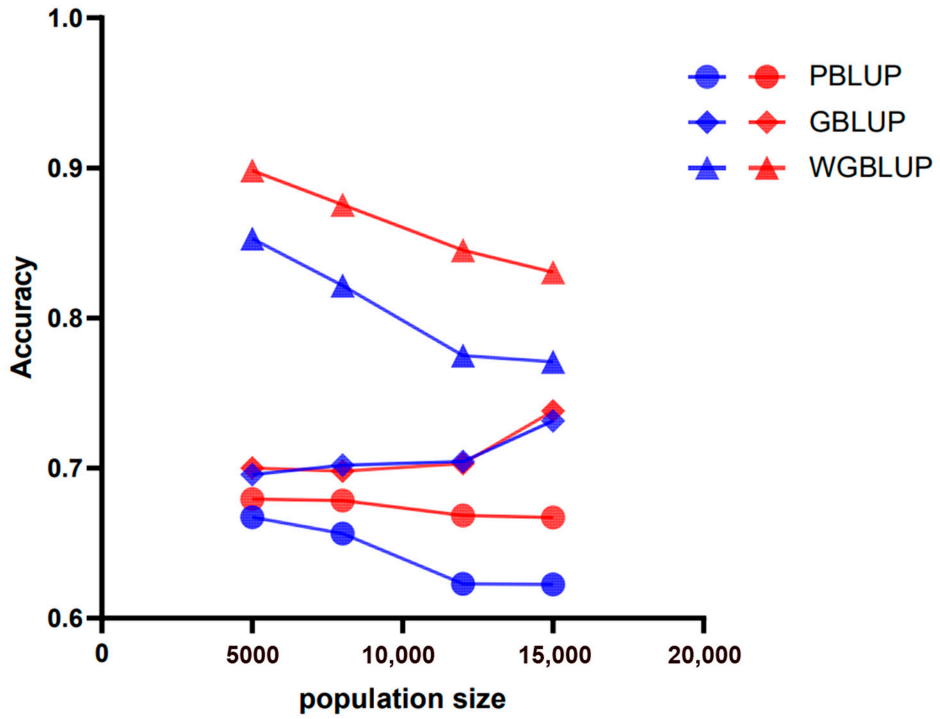

Supplementary Figure S6. Prediction accuracy of Genomic Estimated Breeding Values (GEBVs) for simulated traits with a heritability of 0.7 in breed C using different evaluation methods: PBLUP, GBLUP or WGBLUP. The X-axis represents the number of animals in the reference population, while the Y-axis indicates the predicted accuracy of GEBVs for the simulated traits. The blue and red lines correspond to marker densities of 50 k and 770 k, respectively.

Supplementary Table S1

Accuracies of genomic prediction using PBLUP, GBLUP or WGBLUP procedures under different training populations, with varying levels of heritability and marker densities for breed B.

Population Size	h^2	50 k			770 k		
		PBLUP	GBLUP	WGBLUP	PBLUP	GBLUP	WGBLUP
5000	0.3	0.5373137	0.6657639	0.7052539	0.661721	0.6765822	0.7440888
	0.5	0.5927053	0.7405959	0.7868989	0.7432708	0.7673241	0.8534381
	0.7	0.6470749	0.7814013	0.863098	0.7706468	0.7956605	0.8739026
8000	0.3	0.5791717	0.6590116	0.7355134	0.6663261	0.6782512	0.7430619
	0.5	0.5726825	0.7388189	0.7607198	0.7496312	0.7531426	0.826754
	0.7	0.6101892	0.7765323	0.8114894	0.7338687	0.7830507	0.8294176
12000	0.3	0.5741335	0.7078567	0.727636	0.6756349	0.7047986	0.7586326
	0.5	0.5881436	0.7679576	0.7655882	0.7477593	0.7749613	0.8210688
	0.7	0.5955545	0.8008031	0.7946652	0.7780654	0.8106076	0.8173453
15000	0.3	0.5802694	0.7296113	0.7300001	0.7106253	0.7231496	0.7644497
	0.5	0.592403	0.7854425	0.7660125	0.7462347	0.7876068	0.8131678
	0.7	0.5916221	0.8085625	0.7851947	0.7214303	0.8189681	0.8191987

Supplementary Table S2

Accuracies of genomic prediction using PBLUP, GBLUP or WGBLUP procedures under different training populations, with varying levels of heritability and marker densities for breed C.

Population Size	h^2	50 k			770 k		
		PBLUP	GBLUP	WGBLUP	PBLUP	GBLUP	WGBLUP
5000	0.3	0.6268918	0.6399406	0.72914	0.6225569	0.6361501	0.7515934
	0.5	0.6450285	0.685987	0.7968326	0.6912384	0.692037	0.8504291
	0.7	0.6672837	0.6958102	0.8528879	0.6793339	0.7000565	0.8983289
8000	0.3	0.6143694	0.6369493	0.7164189	0.6319841	0.6309623	0.7387592
	0.5	0.6592215	0.6896186	0.7713919	0.7001958	0.7207194	0.82676
	0.7	0.656368	0.701963	0.8215814	0.6783387	0.6980101	0.875512
12000	0.3	0.5948404	0.6342216	0.6993367	0.6400037	0.6435219	0.7336957
	0.5	0.6144336	0.6906163	0.7435381	0.6778702	0.7006853	0.7981402
	0.7	0.6228097	0.7042935	0.7750303	0.6684452	0.7030898	0.8452694
15000	0.3	0.6162032	0.6647648	0.7125587	0.6587097	0.6643078	0.7459968
	0.5	0.6757691	0.7141493	0.7436045	0.6686287	0.7188344	0.7916278
	0.7	0.6224577	0.7315414	0.7707369	0.6670493	0.7380079	0.8304661
